# Supplementary material for: Study Protocol for Two-Steps Parallel Randomized Controlled Trial: Pre-Clinical Usability Tests for a New Double-Chamber Syringe
Source: Int J Environ Res Public Health. 2020 Nov 12;17(22):8376. doi: 10.3390/ijerph17228376 (PMC7696070; doi:10.3390/ijerph17228376)
Supplement: Supplementary file 1 [file ijerph-17-08376-s001.pdf]

USABILITY QUESTIONNAIRE | MEDICAL DEVICES

This questionnaire aims to evaluate a set of aspects related to Medical Devices (DM), in this particular case, the DUO Syringe. To this end, you are asked to indicate your level of agreement in relation to each of the questions posed below, according to the following scale of response:

1	2	3	4	5	6	7
**Total disagree**	◄	—	—	—	►	**Total agree**

Usefulness items		
(The use of double-chamber syringe for intravenous therapeutics…)		
	1. is useful for my work.	1 2 3 4 5 6 7
	2. facilitates the performance of my tasks.	1 2 3 4 5 6 7
	3. help me to be more effective.	1 2 3 4 5 6 7
	4. help me to be more efficient.	1 2 3 4 5 6 7
	5. achieve everything I would expect it to do.	1 2 3 4 5 6 7
	6. allows me to complete my tasks.	1 2 3 4 5 6 7
	7. allows me to complete my tasks easily.	1 2 3 4 5 6 7
	8. allows me to complete my tasks quickly.	1 2 3 4 5 6 7
	9. allows me a better control on performing my tasks.	1 2 3 4 5 6 7
	10. help me to be more productive in my work.	1 2 3 4 5 6 7
	11. allows me to ensure the patient safety.	1 2 3 4 5 6 7
	12. allows me to answer my needs.	1 2 3 4 5 6 7
Ease of use items		
(The double-chamber syringe for intravenous therapeutics…)		
	13. is ease to use.	1 2 3 4 5 6 7
	14. is simple to use.	1 2 3 4 5 6 7
	15. is user friendly.	1 2 3 4 5 6 7
	16. requires few steps to accomplish my work.	1 2 3 4 5 6 7
	17. allows flexible use according to my needs.	1 2 3 4 5 6 7
	18. doesn't require physical effort to use it.	1 2 3 4 5 6 7
	19. doesn't require mental effort to use it.	1 2 3 4 5 6 7
	20. allows me to complete tasks in a logical sequence.	1 2 3 4 5 6 7
	21. isn't associated with large error possibility in its use.	1 2 3 4 5 6 7
	22. allows me to recover from mistakes quickly and easily.	1 2 3 4 5 6 7

1	2	3	4	5	6	7
**Total disagree**	←				→	**Total agree**

Ease of learning items		
(Concerning double-chamber syringe for intravenous therapeutics…)		
	23. I learned to use it quickly.	1 2 3 4 5 6 7
	24. I learned to use it easily.	1 2 3 4 5 6 7
	25. I easily remember how to use it.	1 2 3 4 5 6 7
	26. I quickly became skilful with it.	1 2 3 4 5 6 7
	27. it isn't necessary too much previous knowledge to use it.	1 2 3 4 5 6 7
	28. it isn't necessary written instructions to use it.	1 2 3 4 5 6 7
Satisfaction / Intention to use items		
(Concerning the possibility to use double-chamber syringe for intravenous therapeutics in future…)		
	29. I will be satisfied with it.	1 2 3 4 5 6 7
	30. I would recommend it to colleagues.	1 2 3 4 5 6 7
	31. it will allowed the performance of my tasks.	1 2 3 4 5 6 7
	32. it will be interesting to the performance of my tasks.	1 2 3 4 5 6 7
	33. I feel I need to have it in my work.	1 2 3 4 5 6 7
	34. it will be pleasant to use.	1 2 3 4 5 6 7
	35. I will feel comfortable in use it.	1 2 3 4 5 6 7
	36. I will feel confident in use it.	1 2 3 4 5 6 7
	37. I will feel security in use it.	1 2 3 4 5 6 7
	38. the dimensions of the device are adjusted.	1 2 3 4 5 6 7
	39. the weight of the device is adjusted.	1 2 3 4 5 6 7
	40. the appearance of the device is adjusted.	1 2 3 4 5 6 7
	41. I will like to use it frequently.	1 2 3 4 5 6 7
	42. it will be easy to adjust it to perform my work.	1 2 3 4 5 6 7
